# Improving open and rigorous science: ten key future research opportunities related to rigor, reproducibility, and transparency in scientific research

**DOI:** 10.12688/f1000research.26594.1

**Published:** 2020-10-14

**Authors:** Danny Valdez, Colby J. Vorland, Andrew W. Brown, Evan Mayo-Wilson, Justin Otten, Richard Ball, Sean Grant, Rachel Levy, Dubravka Svetina Valdivia, David B. Allison

**Affiliations:** 1 Indiana University School of Public Health, Bloomington, IN, 47403, USA; 2 Project TIER, Haverford College, Haverford, Pennsylvania, 19041, USA; 3 Indiana University Purdue University Indianapolis Fairbanks School of Public Health, Indianapolis, IN, 46223, USA; 4 Rachel Levy, Mathematical Association of America, 1529 18th St. NW, Washington, DC, 20036, USA; 5 Indiana University School of Education, Bloomington, IN, 47401, USA

**Keywords:** Meta-Science; Science of Science; Rigor Reproducibility and Transparency (RRT); Workshop;

## Abstract

**Background:** As part of a coordinated effort to expand research activity around rigor, reproducibility, and transparency (RRT) across scientific disciplines, a team of investigators at the Indiana University School of Public Health-Bloomington hosted a workshop in October 2019 with international leaders to discuss key opportunities for RRT research.

**Objective: **The workshop aimed to identify research priorities and opportunities related to RRT.

**Design:** Over two-days, workshop attendees gave presentations and participated in three working groups: (1) Improving Education & Training in RRT, (2) Reducing Statistical Errors and Increasing Analytic Transparency, and (3) Looking Outward: Increasing Truthfulness and Accuracy of Research Communications. Following small-group discussions, the working groups presented their findings, and participants discussed the research opportunities identified. The investigators compiled a list of research priorities, which were circulated to all participants for feedback.

**Results: **Participants identified the following priority research questions: (1) Can RRT-focused statistics and mathematical modeling courses improve statistics practice?; (2) Can specialized training in scientific writing improve transparency?; (3) Does modality (e.g. face to face, online) affect the efficacy RRT-related education?; (4) How can automated programs help identify errors more efficiently?; (5) What is the prevalence and impact of errors in scientific publications (e.g., analytic inconsistencies, statistical errors, and other objective errors)?; (6) Do error prevention workflows reduce errors?; (7) How do we encourage post-publication error correction?; (8) How does ‘spin’ in research communication affect stakeholder understanding and use of research evidence?; (9) Do tools to aid writing research reports increase comprehensiveness and clarity of research reports?; and (10) Is it possible to inculcate scientific values and norms related to truthful, rigorous, accurate, and comprehensive scientific reporting?

**Conclusion: **Participants identified important and relatively unexplored questions related to improving RRT. This list may be useful to the scientific community and investigators seeking to advance meta-science (i.e. research on research).

## Introduction

Rigor, reproducibility, and transparency (RRT) are scientific cornerstones that promote truthful, accurate, and objective science ( McNutt, 2014). In the context of scientific research, rigor is defined as a thorough, careful approach that enhances the veracity of findings ( Casadevall & Fang, 2012). There are several types of reproducibility, which include the ability to evaluate and follow the same procedures as previous studies, obtain comparable results, and draw similar inferences ( Goodman *et al*., 2016; National Academies of Sciences, 2019). Transparency is a process by which methodology, experimental design, coding, and data analysis tools are reported clearly and openly shared ( Nosek *et al*., 2015; Prager *et al*., 2019). Together, these scientific norms represent the best means of obtaining objective knowledge of the world ( Anderson *et al*., 2010; Allison *et al*., 2016). The science concerning these norms is a specific branch of meta-science, or “research on research”, led by scientists who promote these values through the education of early career scientists, identifying areas of concern for scientific validity, and postulating paths toward stronger, more credible science ( Ioannidis *et al*., 2015).

Several factors compete with the pursuit of rigorous, reproducible, and transparent research. For example, the rate of scientific publication has risen dramatically in the last two decades. Although this is indicative of many important scientific breakthroughs ( Van Noorden, 2014), the rate of manuscript retractions due to either researcher error or malfeasance has also increased ( Steen *et al*., 2013). A survey found between 40% and 70% of scientists agreed that factors including fraud, selective reporting, and pressure to publish contribute to the irreproducibility of scientific findings ( Fanelli, 2018). These concerns also have the potential to decrease public trust in science, although research on this question is needed ( National Academies of Sciences, 2017).

Basic and applied science are undermined when scientists fail to uphold high standards of conduct ( Prager *et al*., 2019). Given that many authors have identified issues or concerns in science, the emerging challenge for scholars in this area is to find workable solutions to improve RRT, rather than simply continuing to illustrate problems related to RRT ( Allen & Mehler, 2019). To this end, in October 2019, Indiana University School of Public Health-Bloomington hosted a multidisciplinary meeting of leading scholars to discuss ongoing RRT-related challenges. The purpose of the meeting, which was funded by the Afred P. Sloan Foundation, was to identify new opportunities to advance sound scientific practice, from the early stages of planning a study, through to execution and the communication of findings. This paper presents findings from that meeting.

## Methods

The meeting was structured around three areas:

(1) Improving education & training in RRT.
(2) Reducing statistical errors and increasing analytic transparency.
(3) Looking outward: increasing truthfulness and accuracy of research communications.

### Participants

We invited participants based on prior contributions to RRT research. Participants included representatives from several leading organizations and Indiana University (IU) faculty, staff, and graduate students who were invited to participate in the meeting and proceedings ( Table 1). For their participation in the meeting, invited guests who were not federal employees or IU employees received a $1,000 honorarium.

**Table 1.** List of invited participants and other attendees.

Name	Affiliation	Subgroup
**Invited participants**		
Richard Ball, Ph.D.	Project TIER [Teaching Integrity in Empirical Research]	Improving Education & Training in rigor, reproducibility, and transparency [RRT]
Rachel Levy, Ph.D.	Mathematical Association of America	Improving Education & Training in RRT
Keith Baggerly, Ph.D.	University of Texas	Reducing Statistical Errors and Increasing Analytic Transparency
John Ioannidis, M.D., DSc	METRICS [Meta-Research Innovation Center at Stanford]	Reducing Statistical Errors and Increasing Analytic Transparency
Brian Nosek, Ph.D.	Center for Open Science	Reducing Statistical Errors and Increasing Analytic Transparency
Phillipe Ravaud, M.D., Ph.D.	Paris Descartes University	Looking Outward: Increasing Truthfulness and Accuracy of Research Communications
Machell Town, Ph.D.	Centers for Disease Control and Prevention	Looking Outward: Increasing Truthfulness and Accuracy of Research Communications
Matt Vassar, MBA, Ph.D.	Oklahoma State University	Looking Outward: Increasing Truthfulness and Accuracy of Research Communications
**Indiana University faculty & staff**		
David B. Allison, Ph.D.	Dean of the School of Public Health	Improving Education & Training
Dubravka Svetina, Ph.D.	School of Education	Improving Education & Training
Elizabeth Housworth, Ph.D.	Mathematics	Improving Education & Training
Emily Meanwell, Ph.D.	Social Science Research Commons	Improving Education & Training
Roger Zoh, MS, Ph.D.	School of Public Health	Improving Education & Training
Andrew W. Brown, Ph.D.	School of Public Health	Reducing Statistical Errors and Increasing Analytic Transparency
Stephanie Dickinson, MS	School of Public Health	Reducing Statistical Errors and Increasing Analytic Transparency
Mandy Mejia, Ph.D.	Statistics	Reducing Statistical Errors and Increasing Analytic Transparency
Carmen Tekwe, MS, Ph.D.	School of Public Health	Reducing Statistical Errors and Increasing Analytic Transparency
Evan Mayo-Wilson, DPhil	School of Public Health	Looking Outward: Increasing Truthfulness and Accuracy of Research Communications
Ana Bento, Ph.D.	School of Public Health	Looking Outward: Increasing Truthfulness and Accuracy of Research Communications
Jutta Schickore, Ph.D.	History and Philosophy of Science and Medicine	Looking Outward: Increasing Truthfulness and Accuracy of Research Communications
Jamie Wittenberg, M.B.S, MSLIS	Library System	Looking Outward: Increasing Truthfulness and Accuracy of Research Communications
**Non-presenting attendees**		
Lilian Golzarri Arroyo, MS (IU School of Public Health); Chris Bogert, Ph.D. (IU Applied Pharmacology & Toxicology); Sean Grant, DPhil (IUPUI School of Public Health); Stasa Milojevic, Ph.D. (IU Informatics); Luis Mestre, MS (IU School of Public Health); Justin Otten, Ph.D. (IU School of Public Health); Danny Valdez, Ph.D. (IU School of Public Health); Colby J. Vorland, Ph.D. (IU School of Public Health)		

### Meeting format

The two-day meeting was comprised of nine prepared research talks, moderated panel discussions, and small-group open-forum style sessions related to each of the three previously stated goals.

***Day one.*** On the first day, participants presented 10–12 minute research talks, each of which was followed by a moderated question-and-answer period. Participants discussed questions pertaining to RRT and sought to identify emerging areas of research including novel approaches, testable outcomes, and potential limitations. During the afternoon session, participants were divided into three small-groups to discuss potential research opportunities, moderated by an IU faculty representative charged with compiling notes for record keeping and dissemination.

***Day two.*** On the second day, one representative from each group summarized major points through a brief presentation, which was followed by a question-and-answer session with all participants. This dialogue was intended to clarify ideas raised and to identify fundable research opportunities. The meeting concluded with a call to action by the Dean of the School of Public Health-Bloomington and Co-Principal Investigator of the project (DA), to continue promoting interdisciplinary RRT Science.

## Results

### Subgroup 1: improving education & training in RRT

We asked the first subgroup to discuss research opportunities related to implementing and testing RRT-guided academic curricula. The group identified elements of current undergraduate and graduate education that contribute to problematic data practices, including possible underlying causes and potential solutions (see Table 2). Three primary education-related questions guided the discussion:

**Table 2.** Summary of group discussion.

Group	Question	Challenges ^1^
**Subgroup 1:** improving education & training in rigor, reproducibility, and transparency (RRT)	*1. Can RRT-focused statistics and mathematical modeling courses* *improve statistical practice?*	1. It would be difficult to isolate and to evaluate the effects of changes to existing curricula.
		2. Proximal measures related to technical skills might not translate into improved research practices.
	*2. Can specialized training in scientific writing improve transparency?*	1. Writing is an abstract science, which would make measuring outcomes challenging.
		2. There are currently limited existing graduate level curricula that pertain exclusively to writing.
	*3. Does modality affect the efficacy of RRT-related education?*	1. Feasibility concerns including, cost, time, and other additional resources needed to facilitate an intervention.
		2. Examining heterogeneity requires large and diverse populations, and is practically difficult.
**Subgroup 2:** reducing statistical errors and increasing analytic transparency	*4. Can automation help identify errors more efficiently?*	1. Automation may be technically possible for only certain types of errors.
		2. New programs intended to automate error correction require a certain level of computer programming expertise.
	*5. What is the prevalence and impact of errors?*	1. It would be difficult to generalize the prevalence of errors, because many common errors have field-specific names.
		2. Assessing the impact of errors is largely subjective, unless strict guidelines are agreed upon and adopted.
	*6. Do error prevention workflows reduce errors?*	1. It would be difficult to determine if workflows are entirely responsible for reduced error and improved research practice.
		2. It may be challenging to identify generalizable workflows that logically function across disciplines.
	*7. How do we encourage post-publication error correction?*	1. It would be difficult to implement standard post-publication error correction guidelines that function effectively across disciplines.
		2. There is a hesitancy to embrace error correction as a normal component of the editorial process.
**Subgroup 3:** looking outward: increasing truthfulness and accuracy of research communications	*8. How does 'spin' in research communication affect stakeholders'* *understanding and use of research evidence?*	1. The effects of spin in controlled research settings might not generalize to real-world decisions.
		2. Reviewing and categorizing text is both subjective and time consuming.
	*9. Do tools to aid writing research reports increase the* *comprehensiveness and clarity of research reports?*	1. Although tools could be developed for testing, implementation challenges could mitigate their effectiveness in practice.
		2. Previous guidelines have had minimal impact on reporting quality.
	*10. Is it possible to inculcate scientific values and norms related to* *truthful, rigorous, accurate, and comprehensive scientific reporting?*	1. There are few model interventions to form self-identity.
		2. There may be limited opportunities and enthusiasm to integrate values-based education in classes that focus on technical skills.

^1^We present here only two of the most salient challenges.

(1) Can RRT-focused statistics and mathematical modeling courses improve statistical practice?
(2) Can specialized training in scientific writing improve transparency?
(3) Does modality affect the efficacy of RRT-related education?

With respect to each question the existing and entrenched practices, feasibility of change, and proper audience for interventions were discussed.

**1. Can RRT-focused statistics and mathematical modeling courses improve statistical practice?**

Incorrect analyses are some of the most common, preventable errors in science ( Resnik, 2012). Scholars attribute mistakes to gaps in statistics education ( Thompson, 2006). With the rise in data science as a component of scientific exploration, students need more exposure to evidence-based pedagogical approaches to statistics and mathematical modeling ( GAISE, 2016; GAIMME, 2016, and NASEM, 2018). Many introductory data science courses include topics from statistics (e.g., contingency tables [chi-square tests], multiple regression, analysis of variance, and the broader general linear model) ( Gorsuch, 2015), as well as mathematical modeling approaches and computational algorithms. These topics can be reframed through an RRT lens as modules/domains within existing mathematics or data-science courses or structured as new data-driven courses entirely.

Indeed, participants noted that to improve RRT practices, there are opportunities to design new courses with a direct RRT focus at the undergraduate, graduate and postdoctoral levels ( Willig *et al*., 2018). Courses could include modules related to the identification of errors in published research, proposing solutions to these errors, addressing real-world contexts and demonstrating the importance of careful methodological decision-making ( Peng, 2015). Specific assignments could test for and reinforce RRT principles, such as research compendia (i.e. sharable electronic folders containing code, and other exploratory information to validate reported results) ( Ball & Medeiros, 2012; King, 1995; Stodden *et al*., 2015), workflows, which are described later in this paper, and other research projects related to communication and computational reproducibility. The learning practices could be assessed to ensure that students appropriately apply concepts rather than demonstrate rote-formula memorization ( Thompson, 2002; Ware *et al*., 2013). Integrating learning into stages of education where students are concurrently engaged in research can help improve both retention and transfer of the RRT ideas to future scientific settings.

**2. Can specialized training in scientific writing improve transparency?**

Clear scientific writing is necessary to reproduce and build on research findings. To facilitate better writing, scholars have developed curricula to help academics improve writing practice and quality (e.g., Goodson, 2016). However, many academic writing programs focus on personal habit building and development of linguistic mechanics to craft more powerful prose ( Elbow, 1998; Kellogg & Whiteford, 2009). In such courses, RRT-related dimensions of writing (such as writing transparently or minimizing ‘spin’) may not be emphasized. Thus, the subgroup discussed how existing writing curricula could incorporate RRT principles, what new writing courses guided by RRT would entail, and research opportunities to test the efficacy of new writing curricula.

Participants identified several RRT-specific writing principles and discussed how a deeper understanding of the extent to which writing and research are intertwined may increase transparency. Examples included learning about methodological reporting guidelines, writing compelling post-publication peer reviews, and other transparent writing practices. The group also discussed how courses could be developed or redesigned specifically to center on RRT principles. One theme of the discussion was the need for rigorous testing of student learning outcomes associated with novel writing content. However, a primary concern was the identification of the appropriate outcome measures for writing-specific interventions ( Barnes *et al*., 2015) given the subjective and nebulous nature of constructs like writing quality, individual improvement, and writing-related self-efficacy.

**3. Does modality affect the efficacy of RRT-related education?**

Another research opportunity discussed by the subgroup related to instructional modality, which refers to the manner in which a curriculum or intervention is experienced by the learner ( Perry & Pilati, 2011). These may include traditional face-to-face instruction, synchronous or asynchronous online meetings/trainings, and various hybrid formats ( Beall *et al*., 2014). Understanding the relative benefits of each modality is important in choosing an appropriate intervention. Indeed, educational needs vary among learner groups; for example, what is most effective for undergraduate students may not be effective or feasible for post-doctoral researchers with full-time professional commitments. Broad research questions identified by the group included:

a) What modalities exist beyond face-to-face, online, or hybrid instruction?;
b) How can technology push modality beyond online courses and other Massive Open Online Course formats?
c) Which modality is most effective and among which audiences?

In the context of previously discussed coursework in statistics and writing, participants explored the strengths and weaknesses of various modalities and how interventions could be conducted to test them empirically. There are logistical considerations, such as cost, space, and faculty time, that further complicate the feasibility of these interventions. For example, a face-to-face intervention may offer more tailored instruction to individual learners, while an online intervention may better deliver content to a wider audience. Thus, the subgroup identified several areas for future research, including comparisons of student learning across modalities, strategies for scaling educational content to institutional constraints, and the moderating effects of learner demographics on intervention efficacy.

### Subgroup 2: reducing statistical errors and increasing analytical transparency

Errors are “actions or conclusions that are demonstrably and unequivocally incorrect from a logical or epistemological point of view” ( Brown *et al*., 2018). Despite the adage that science is self-correcting, uncorrected errors are prevalent in the scientific literature ( Brown *et al*., 2018; Ioannidis, 2012). Subgroup 2 discussed questions related to reducing and mitigating such errors, including:

(4) Can automation help identify errors more efficiently?;
(5) What is the impact of errors within disciplines?;
(6) Do standardized procedures (i.e., workflows) prevent errors?
(7) How do we encourage post-publication error correction?

The costs and benefits associated with each question were also discussed (see Table 2).

**4. Can automation help identify errors more efficiently?**

Various automated and manual methods have been developed and applied to assess analytic inconsistencies, statistical errors and improbabilities, and other errors (e.g., Anaya, 2016; Baggerly & Coombes, 2009; Brown & Heathers, 2017; Georgescu & Wren, 2018; Labbé *et al*., 2019; Monsarrat & Vergnes, 2018). An increase in automation (i.e., producing more user-friendly tools and algorithms) has the potential for surveilling the prevalence, prevention, and correction of errors. However, more work is needed to determine the most efficient use of such tools, including their collective abilities to detect field-specific issues that require subject matter expertise ( Lakens & Debruine, 2020). For example, the automatic recomputation of some p-values is possible using the program ‘Statcheck’, but only for articles that utilize the American Psychological Association’s (APA) in-text citation style for statistical reporting ( Nuijten *et al*., 2017). Other examples require statistical ratios ( Georgescu & Wren, 2018), or integer-based data and sample sizes (e.g., Brown & Heathers, 2017; Heathers *et al*., 2018), which are both challenging to automate and not recurrent across all fields.

Automated error detection is currently limited to a narrow range of errors. Other types of errors might be detected by careful readers, such as the ignoring of clustering in cluster-randomized trials ( Brown *et al*., 2015; Heo *et al*., 2018), misinterpretation of differences in nominal significance, and post-hoc fallacies ( Brown *et al*., 2019; George *et al*., 2016). The subgroup discussed opportunities to define, and possibly automate, diagnostic checklists, advanced natural language processing, or other computational informatics approaches that would facilitate the detection of these errors. These novel automated measures could be tested empirically for effectiveness.

**5. What is the prevalence and impact of errors?**

Different errors will have varying impacts on study conclusions. While some errors can be easily corrected and reported, some fundamentally invalidate study conclusions. Some general statistical errors have occurred repeatedly across disciplines for decades (e.g., mistaken differences due to “regression to the mean” since at least 1886 [ Thomas *et al*., 2020] and “differences in nominal significance” for decades [ Altman, 2002; Thompson, 2002]). Automated methods, such as those outlined above, have been used almost exclusively to illuminate problems but not necessarily correct them ( Georgescu & Wren, 2018; Monsarrat & Vergnes, 2018; Nuijten *et al*., 2017).

To achieve the goal of error reduction, one must first know how pervasive errors are. Yet, it remains challenging to generalize the detection and correction of scientific errors across disciplines because of field specificity (i.e. the unique nuances and methodological specificities inherent to a specific field of study) ( Lohse *et al*., 2020), the various terminologies used for describing the same models (e.g. ‘Hierarchical Linear’ models vs ‘Multilevel’ models), as well as the seeming need to repackage the same problem as new disciplines arise (e.g. ongoing multiple comparison issues raised anew with the advent of genome-wide association studies, microarray, microbiome, and functional magnetic resonance imaging methods). Thus, this subgroup discussed the value of longitudinal, discipline-specific error surveillance and error frequency estimation to collect empirical evidence about error rate differences among disciplines. Other issues discussed were the identification of better prevalence estimates across fields, and how simulation studies can modify our confidence in the understanding of the prevalence of errors and their generalizability across disciplines.

**6. Do error prevention workflows reduce errors?**

Workflows are the various approaches for accomplishing scientific objectives, usually expressed as tasks and dependencies ( Ludäscher *et al.,* 2009). The implementation of clear, logical workflows can potentially prevent errors and improve research transparency. Workflows may be of value to catch errors at various stages of the research process, from planning, to data collection and handling procedures, and reporting/manuscript screening ( Cohen-Boulakia *et al*., 2017). Error detection processes within scientific workflows may serve as mechanisms to prevent errors before publication, akin to how text duplication software (e.g. iThenticate) is used prophylactically to catch inadvertent plagiarism. Separately, some research groups implement workflows that require two independent scientists to verify data, analyses, and statistical reporting prior to manuscript publication, with at least one of those individuals being a professional statistician ( George *et al*., 2016). A similar workflow is to establish “red teams”, consisting of methodologists, statisticians, and subject-matter experts, to critique the study design and analysis for errors, offering incentives akin to “bug bounty” programs in computer software development ( Lakens, 2020).

The development and dissemination of research workflows could be modeled after those outlined above, or in other ways such as the use of checklists to complete work systematically. Registrations, reporting guidelines, and other workflow approaches essentially serve as checklists of the plan for a study and what should be reported. Although this subgroup agreed about the importance of preventive versus post-publication workflows and integration of automated methods to detect errors, questions regarding their efficacy remained. For example, how might workflows be generalized across academic disciplines? At what level should standardizing data collection and handling be taught to scientists to maintain data provenance (e.g. Long, 2009)? And can workflows be tested empirically? What is the cost of automated versus manual workflows, versus none at all, at detecting and preventing errors? How do workflows impact productivity?

**7. How do we encourage post-publication error correction?**

Science cannot self-correct without processes that facilitate correction ( Firestein, 2012). Unfortunately, errors in science may be tied with perceived reputation costs, yet it is unclear that correcting errors actually harms a researcher’s reputation ( Azoulay *et al*., 2015). Thus, destigmatizing error correction and likewise embracing the importance of scientific failures may be of value for individual scientists and editors overseeing content through the peer-review process ( Teixeira da Silva & Al-Khatib, 2019). Journals and their editors, as gatekeepers of science, are key stakeholders in this culture shift. They may also require practical guidelines to facilitate judgement-free corrections that would be acceptable to editors and reviewers.

Error correction should be done in a fair and efficient manner (e.g., Vorland *et al*., 2020). Although there are several existing standards for publication ethics and norms (e.g., Committee on Publication Ethics [COPE], and the International Committee of Medical Journal Editors [ICMJE]), few have been tested empirically. The subgroup debated how journals and their editors could be part of empirically tested trials on best approaches to facilitate correction and minimize the incurring of additional costs. For example, based on our experiences, journals have few procedures for handling errors separate from typical scholarly dialogue. We believe it is important to examine which procedures are more efficient and fair to authors, whether such procedures can be standardized to enable editors to handle different types of errors consistently and transparently, whether correction mechanisms are sufficient or require additional innovation (e.g. retraction and republication is sufficient or versioning), and how authors can be supported and encouraged in the process. Three such costs that require further study include the actual cost of post-publication error correction across all parties involved (e.g. page charges, salary), how those costs to the scientific enterprise compare to implementing prevention strategies, and the cost-benefit of salvaging a publication containing an error depending on the quality of the collected data versus simply retracting.

### Subgroup 3 - looking outward: increasing truthfulness and accuracy of research communications

The third working group discussed opportunities for research related to research reporting and dissemination, primarily highlighting the importance of accuracy and truthfulness when communicating research findings (see Table 2). Specifically, this group identified research opportunities tied to the following questions:

(8) How does ‘spin’ in research communication affect stakeholders’ understanding and use of research evidence?
(9) Do tools to aid writing research reports increase the comprehensiveness and clarity of research reports?
(10) Is it possible to inculcate scientific values and norms related to truthful, rigorous, accurate, and comprehensive scientific reporting?

**8. How does “spin” in research communication affect stakeholders’ understanding and use of research evidence?**

In addition to conducting research rigorously, investigators should describe their research comprehensively and interpret their findings by balancing the strengths and limitations of their methods and results ( Brown *et al.,* 2017). By contrast, researchers might ‘spin’ their results through misleading reporting, misleading interpretation, and inappropriate extrapolation ( Fletcher & Black, 2007; Yavchitz *et al*., 2016). Some evidence suggests that spin is common in reports of clinical trials and meta-analyses ( Boutron *et al*., 2019; Lazarus *et al*., 2015) and that authors in a variety of research disciplines often draw inappropriate causal inferences ( Bleske-Rechek *et al*., 2015; Casazza *et al*., 2013; Chiu *et al*., 2017; Knight *et al*., 1996; Ochodo *et al*., 2013). Moreover, spin in popular media (e.g., newspapers) appears to stem from spin in scientific reports (e.g., journal articles) and associated press releases ( de Semir *et al*., 1998; Schwartz *et al*., 2012; Schwitzer, 2008).

Spin is unscientific, and could have implications for policy and practice ( Adams *et al*., 2016; Boutron *et al*., 2019; Matthews *et al*., 2016). Workshop participants discussed the need for more evidence to determine whether and how spin in scientific reports affects other stakeholders such as healthcare and social service providers, service users, policymakers, and payers. Evidence concerning the ways in which stakeholders use and interpret research evidence could inform future efforts to improve research communication ( Boutron *et al*., 2019; Lazarus *et al*., 2015).

**9. Do tools to aid writing research reports increase the comprehensiveness and clarity of research reports?**

Research reports (e.g., journal articles) should describe what was done and what was found ( von Elm *et al*., 2007). Stakeholders need comprehensive and accurate information about research methods and results to assess risk of bias, interpret the generalizability of study results, and reproduce the conditions (e.g., interventions) described ( Moher *et al*., 2011). Reporting guidelines describe the minimum information that should be included in reports of different types of research, yet much evidence suggests that scientific reports do not include this information (e.g., Grant *et al*., 2013). Some tools to help authors write better reports have been developed, such as the consort-based web tool (COBWEB) ( Barnes *et al*., 2015); some preliminary evaluations suggest that these tools could help authors write better reports.

Workshop participants identified a need for research to develop and to test tools that could help authors write reports that adhere to existing guidelines. Some tools could be used when writing scientific manuscripts ( Turner *et al*., 2012) while other tools could be used in graduate education (e.g. class assignments, dissertation writing) or continuing education. Guidelines designed to increase authors’ and reviewers’ knowledge of reporting requirements are not commonly adhered to and, thus, have minimal impact on reporting quality ( Capers *et al*., 2015). Participants emphasized the need for new interventions and implementation research that promote guideline adherence.

**10.** **Is it possible to inculcate scientific values and norms related to truthful, rigorous, accurate, and comprehensive scientific reporting?**

In the 1940s, Robert Merton proposed that communism/communality, universalism, disinterestedness, and organized skepticism constitute the ethos of modern science ( Merton, 1942). As the National Research Council stated in their report “Scientific Research in Education”, these fundamental principles are enforced by the community of researchers that shape scientific understanding ( Shavelson & Towne, 2003). Evidence suggests that most scientists endorse these positive values and norms, but fewer scientists believe that their colleagues behave in accordance with these positive norms ( Anderson *et al*., 2007). Better incentives ( Begley *et al*., 2017; Fanelli, 2010; Nosek *et al*., 2012) and better methods for detecting scientific errors, might improve scientific practice and communication; yet fundamentally, we will always have to place some trust in the veracity of our fellow scientists ( Jamieson *et al*., 2017).

Participants agreed that ethics and responsibility are vital across scientific disciplines, yet graduate research often neglects the philosophy of science and the formation of professional identity as a scientist. Instead, training tends to focus on the technical skills needed to conduct experiments and analyze data in specific disciplines ( Bosch, 2018; Bosch & Casadevall, 2017). Technical skills are essential to produce good science; to apply them ethically and responsibly, however, it is paramount that scientists also endorse scientific values and norms. Participants identified a need for research to determine how these scientific values could be inculcated in scientists and how scientists should be taught to enact those values in their research.

## Conclusion

Scientists slow the pursuit of truth when research is not rigorous, reproducible, or transparent ( Collins & Tabak, 2014). To improve the state of science, RRT leaders have long raised concerns about many of the current challenges the scientific enterprise faces by identifying novel strategies intended to uphold and improve scientific validity. Discussions among RRT leaders at Indiana University Bloomington reinforce the value and importance of promoting accurate, objective, and truthful science. The proposal, execution, and evaluation of the ideas presented herein showcases how the collective and interdisciplinary efforts of those investing in the future of science can solve problems in unique and exciting ways.

## Data availability

No data are associated with this article.

All participants have provided their permission to be named in this article.
