# Supplementary material for: Improving open and rigorous science: ten key future research opportunities related to rigor, reproducibility, and transparency in scientific research
Source: F1000Res. 2020 Oct 14;9:1235. [Version 1] doi: 10.12688/f1000research.26594.1 (PMC7898357; doi:10.12688/f1000research.26594.1)
Supplement: Peer review history

# Peer review: Reviewer response for version 1

**DOI:** 10.5256/f1000research.29358.r78363

**Published:** 2021-02-19

**Authors:** Sheenah M Mische

**Affiliations:** 1 Department of Pathology, New York University (NYU) Langone Medical Center, New York, NY, USA

This manuscript is a concise summary of a two day workshop held at Indiana University School of Public Health - Bloomington on identifying key opportunities for rigor, reproducibility & transparency (RRT) in research. This is not a research report, rather a report on the status of scientific research. The meeting attendance was invitation only and IU faculty staff and graduate students were joined by invited participants with recognized expertise in RRT, and reflected in the extensive references. Opportunities were focused in three key areas: 1) education and training, 2) reducing statistical errors while increasing analytical transparency and 3) improving transparency (truthfulness) and accuracy of research communications to promote accurate, objective, and truthful science. The article reads well with a focus on biomedical research.

**Specific Comments:**

 This manuscript provided an excellent summary of numerous and important challenges facing the research enterprise. There were no applicable outcomes or solutions. Of particular note:

1. Education and training: instructional modality, and understanding the relative benefits of various hybrid formats: there is no debate on the importance of RRT education and training. Both formal and informal forums are critical to research-integrity issues. Bringing scientific integrity issues into the open provides practical guidance for everyone from graduate students to faculty members. Faced with the pandemic, we all have adapted modalities to virtual platforms, emphasizing the importance of instruction regardless of format. Furthermore, formal instruction in rigorous experimental design and transparency is now required for NIH training, career development, and fellowship applications.
2. Reducing statistical errors while increasing analytical transparency and 3) improving transparency (truthfulness) and accuracy of research communications: sharing knowledge is what drives scientific progress—each new advance or innovation in biomedical research builds on previous observations. Experimental reports must have sufficient information to validate the original results and be verified by other researchers to be broadly accepted as credible by the scientific community. While statistics is a necessary for data interpretation by clinical researchers, psychologists, and epidemiologists whose conclusions depend wholly on statistics, the interpretation of data in papers published in the biological sciences does not always require sophisticated statistical analyses; rather, diligent data reporting and transparency is essential.

**Conclusion:**

 The authors summarize with “proposal, execution, and evaluation of the ideas presented herein showcases how the collective and interdisciplinary efforts of those investing in the future of science can solve problems in unique and exciting ways”. While appreciating this forward looking statement, the message is clear: the issue of reproducibility in science is complex and will continue to be debated and discussed in workshops such as this manuscript describes in the coming years. In response to well-publicized allegations of the inability to reproduce published biomedical research there have been numerous declarations of the components of trustworthy research and research integrity such as the Singapore Statement in 2010, the Montreal Statement in 2013, the Hong Kong Principles in 2019 and the European Code of Conduct for Research Integrity in 2017, and U.S. NIH and NSF Federal RRT policies. Ultimately we are all responsible for careful assessment of the rigor of the prior research, rigorous experimental design for robust and unbiased results by application of the scientific method; consideration of relevant biological variables and authentication of key biological and/or chemical resources used to conduct research and the use of numerical identifiers and the RRID syntax to improve communication of critical experimental details within the research community and to the public.

Is the topic of the opinion article discussed accurately in the context of the current literature?

Yes

Are arguments sufficiently supported by evidence from the published literature?

Yes

Are all factual statements correct and adequately supported by citations?

Yes

Are the conclusions drawn balanced and justified on the basis of the presented arguments?

Yes

Reviewer Expertise:

Pathology, Technology, Shared Research Resource

I confirm that I have read this submission and believe that I have an appropriate level of expertise to confirm that it is of an acceptable scientific standard.

# Peer review: Reviewer response for version 1

**DOI:** 10.5256/f1000research.29358.r78351

**Published:** 2021-02-18

**Authors:** Christopher A Mebane

**Affiliations:** 1 Idaho Water Science Center, US Geological Survey, Boise, Idaho, USA

The article “Improving open and rigorous science....” is a report out on a workshop intended to make recommendations on improving rigor, reproducibility, and transparency (RRT) in interdisciplinary science. The idea of peer reviewing a workshop report is a bit of a curious assignment. What’s a reviewer to say?  No, those weren’t the best topics to debate at your workshop, please reconvene and discuss something else? Raise questions about whether the article faithfully reports the workshop deliberations and consensus, when the reviewer wasn’t there?  As such this review is rather limited. The article reads well and has clearly been well vetted by the authors. The workshop and paper are interdisciplinary, although the focus is strongly slanted toward biomedical research and the health sciences.

**Not all errors are mistakes**

 My only criticism of substance is the use of the term “statistical errors.” Consider replacing it with “statistical mistakes” throughout the manuscript.  In many fields, including mine (environmental science), the word “error” could refer to variability in the data, such as “the standard error of the mean.”  In other contexts, the word error is often used to describe the limits of precision. DNA and cells replicate with small errors, which over time lead to aging and senescence. In analytical chemistry, deviations from instrument values for calibration or quality control samples may be termed measurement error. Measurement error might refer to the inherent limits of a sensor in the instrument or the combined errors of the method. For example, in a bathymetric survey, errors accrue from inherent limits in the measuring distance as a function of sound through water, temperature changes in the water introduce error, a breeze adding motion to the boat introduces error, plants growing on the bottom muddy the signal increasing error, imprecision in the Earth’s spheroid and canyon walls interfere with the GPS, and on and on. The hydrologist tries to reflect the accumulated error with a margin of error statement on overall accuracy.  Those are examples of error – something the scientist always seeks to reduce and to accurately report the uncertainties associated with measurements, modeling, etc., but the presence of error is unavoidable. A mistake on the other hand is a blunder. Attaching the bathymetric sensor backwards, entering the wrong units into the calculations, using a long-wave, deep ocean sensor in shallow water, using the wrong datum, using a poorly suited method, neglecting calibrations, .... Just as with statistical mistakes, the topic of the argument, while there are often different appropriate methods of measurement for just about any scientific setting, some controversial or debatable methods, and some that are just plain wrong.  The focus of the authors is on the latter – helping scientists avoid statistical blunders that are just plain wrong. I strongly urge you to call these “statistical mistakes” which is less ambiguous than “errors.”  There are supposed to be interdisciplinary RRT recommendations.

**Minor suggestions**

 p7., in subsection titled “ *5. What is the prevalence and impact of errors,*” I thought the second paragraph was particularly dense and probably impenetrable to those not already in the know:

 :

*“Thus, [Subgroup 2] discussed the value of longitudinal, discipline-specific error surveillance and error frequency estimation to collect empirical evidence about error rate differences among disciplines. Other issues discussed were the identification of better prevalence estimates across fields, and how simulation studies can modify our confidence in the understanding of the prevalence of errors and their generalizability across disciplines.”*

 I think if you could expand on these points with some examples or examples with citations, readers might have better understanding of what is being recommended.

 That’s all. This was a tightly written report out of the workshop. Thank you for considering my rant about mistakes versus errors, where depending on the field and context, the latter is often a neutral descriptor of uncertainty.

Is the topic of the opinion article discussed accurately in the context of the current literature?

Yes

Are arguments sufficiently supported by evidence from the published literature?

Yes

Are all factual statements correct and adequately supported by citations?

Yes

Are the conclusions drawn balanced and justified on the basis of the presented arguments?

Yes

Reviewer Expertise:

I am an environmental scientist who has published on related topics of research rigor, bias, and transparency in the environmental sciences.

I confirm that I have read this submission and believe that I have an appropriate level of expertise to confirm that it is of an acceptable scientific standard.

# Peer review: Reviewer response for version 1

**DOI:** 10.5256/f1000research.29358.r77096

**Published:** 2021-01-28

**Authors:** Judith A Hewitt

**Affiliations:** 1 Office of Biodefense, Research Resources and Translational Research, Division of Microbiology and Infectious Diseases, National Institute of Allergy and Infectious Diseases, National Institutes of Health, Bethesda, MD, USA

This is a concise and well written summary of a meeting of ~30 people on the vitally important topic of rigor, reproducibility & transparency. The meeting discussion questions were very well formulated, though with the small size of the meeting and the limited number of invited participants outside of the university host, it is difficult to say whether the discussions, presented in a very succinct format of key challenges, is representative of all of the issues or viewpoints on the topic. Nevertheless, this appears to have been a good discussion that raised significant challenges. I would have preferred to see a bit more focus on solutions, as the challenges raised are all daunting.

**Specific Comments:**

**Introduction**:

 Regarding the statement that 40-70% of scientists agreed on factors contributing to irreproducibility, the original citation be used, (Baker, 2016; added ^1^). Also, reference to the funder for the meeting is very much appreciated - but it is "Alfred P Sloan" not "Afred". In the last sentence, ""through to execution" is unwieldy - either "through" or "to" works but no need for both.

**Methods**:

 I very much appreciate the list of participants and acknowledgement of honorariums - kudos on the transparency! I also appreciate knowing who participated in the small groups, but it would have been nice to see the agenda or titles of the Day One research presentations. Were those research or meta-research presentations? Also, "small-groups" should not be hyphenated, in fact you could just say three groups and let the reader come to their own conclusion about size; "breakout" is another useful term.

**Results:**

 Subgroup 1, first paragraph: the following wording could be more precise by changing "three primary education-related questions" (where primary modifies education and not questions) to "three primary questions, education-related," or something similar. Precision of language is one of the articulated goals of training and communication in this article!

 Q5, 2nd paragraph: I disagree with the first sentence, "To achieve the goal of error reduction, one must first know how pervasive errors are." I think any reduction in errors is a win, even without understanding the entire landscape, and needing to fully understand the landscape before attempting solutions is just kicking the can down the road. It's the "measurement" of error reduction or assessing progress toward a particular goal (which is not articulated) that requires knowing the pervasiveness first, and I agree that is extremely difficult to measure.

 Q7, 2nd paragraph, last sentence: I question whether understanding "salary" costs of error correction is a valid pursuit, whether it's a case of pay now or pay later; page charges are a different matter.

**Conclusion:**

 Since the Methods section stated that the meeting ended with a "call to action" to continue promoting interdisciplinary RRT science, I wonder if that call to action is accurately summarized? I found a great summary of the discussion but didn't walk away with a clearly articulated call to action in the very brief conclusion.

**General Comments**:

 I tend to agree that the challenges are many and difficult, though the small group discussions are distilled down to two challenges per question. They are mainly framed in negative terms, which is hard to read as a "call to action" without more detail. Nonetheless, the challenges raised are important and should be addressed, I'm just left scratching my head on what the next step is for many of these, given how they are stated.

 I note that many of the references are from participants at the meeting, which may reflect the meeting content (difficult to judge without seeing the agenda), but does not necessarily instill in others an unbiased approach; this is perhaps a limitation of a small-meeting-by-invitation and could be formally recognized in the paper. This is not a value judgement on the references, indeed there is some balance, but it is a selected view that focuses on the meeting participants.

Is the topic of the opinion article discussed accurately in the context of the current literature?

Yes

Are arguments sufficiently supported by evidence from the published literature?

Partly

Are all factual statements correct and adequately supported by citations?

Yes

Are the conclusions drawn balanced and justified on the basis of the presented arguments?

Partly

Reviewer Expertise:

infectious diseases; animal models of infectious diseases; translational research; scientific rigor; reproducibility

I confirm that I have read this submission and believe that I have an appropriate level of expertise to confirm that it is of an acceptable scientific standard.

## References

[1] : 1,500 scientists lift the lid on reproducibility. *Nature*.2016;533(7604) : 10.1038/533452a 452-454 10.1038/533452a 27225100
